# Negative Effects of High Glucose Exposure in Human Gonadotropin-Releasing Hormone Neurons

**DOI:** 10.1155/2013/684659

**Published:** 2013-12-31

**Authors:** Annamaria Morelli, Paolo Comeglio, Erica Sarchielli, Ilaria Cellai, Linda Vignozzi, Gabriella B. Vannelli, Mario Maggi

**Affiliations:** ^1^Section of Anatomy and Histology, Department of Experimental and Clinical Medicine, University of Florence, 50134 Florence, Italy; ^2^Section of Sexual Medicine and Andrology, Department of Experimental and Clinical Biomedical Sciences, University of Florence, 50134 Florence, Italy; ^3^Centro Interuniversitario di Ricerca sulle Basi Molecolari della Malattie della Riproduzione (CIRMAR), 20122 Milan, Italy

## Abstract

Metabolic disorders are often associated with male hypogonadotropic hypogonadism, suggesting that hypothalamic defects involving GnRH neurons may impair the reproductive function. Among metabolic factors hyperglycemia has been implicated in the control of the reproductive axis at central level, both in humans and in animal models. To date, little is known about the direct effects of pathological high glucose concentrations on human GnRH neurons. In this study, we investigated the high glucose effects in the human GnRH-secreting FNC-B4 cells. Gene expression profiling by qRT-PCR, confirmed that FNC-B4 cells express GnRH and several genes relevant for GnRH neuron function (KISS1R, KISS1, sex steroid and leptin receptors, FGFR1, neuropilin 2, and semaphorins), along with glucose transporters (GLUT1, GLUT3, and GLUT4). High glucose exposure (22 mM; 40 mM) significantly reduced gene and protein expression of GnRH, KISS1R, KISS1, and leptin receptor, as compared to normal glucose (5 mM). Consistent with previous studies, leptin treatment significantly induced GnRH mRNA expression at 5 mM glucose, but not in the presence of high glucose concentrations. In conclusion, our findings demonstrate a deleterious direct contribution of high glucose on human GnRH neurons, thus providing new insights into pathogenic mechanisms linking metabolic disorders to reproductive dysfunctions.

## 1. Introduction

The hypothalamic-pituitary-gonadal (HPG) axis is finely regulated at a central level by the activity of GnRH neurons, a peculiar hypothalamic neuronal subpopulation, comprising few cells (800–2000 cells in the adult brain) scattered within the preoptic area (POA) of the hypothalamus [1, 2]. The anatomical position of GnRH neurons makes them especially vulnerable to peripheral nutrient changes, due to the close proximity to the blood brain barrier (BBB), within the third ventricle [3]. Moreover, recent findings demonstrated that a subpopulation of GnRH neurons projects dendrites in regions outside the BBB, where they may directly sense molecules circulating in the bloodstream, therefore extending the range of factors that are integrated by these neurons for the control of the reproductive axis [4].

Over the past years, compelling experimental evidences have deciphered several mechanisms through which peripheral signals and neuroendocrine pathways are integrated and conveyed to finally regulate GnRH neuron function. In particular, metabolic hormones, including leptin, insulin, ghrelin, and polypeptide XX, may regulate GnRH neuron activity and thereby the HPG axis [5]. In addition to peripheral hormones, novel central mediators responsible for relaying such metabolic messages to centers governing reproduction have been identified. The most recent data from experimental animals indicate a central role played by the kisspeptin/KISS1R system in mediating a range of metabolic inputs known to regulate GnRH secretion ([6–8], for reviews).

Derangements of the HPG axis are often associated with metabolic disorders. In the male population, hypogonadism, a frequent condition in middle-aged and elderly subjects [9], affects patients with type two diabetes mellitus (T2DM) more frequently than subjects without [10–12]. In T2DM patients androgen deficiency is associated with inappropriately normal or even low plasma concentrations of the pituitary gonadotropins—LH and FSH—[13–15] indicating hypothalamic defects and/or impaired pituitary response to GnRH. A normal LH and FSH response to GnRH has been demonstrated in subjects with T2DM, suggesting a hypothalamic rather than a pituitary defect [16]. However, the pathogenic mechanism underlying a relationship between hypogonadotropic hypogonadism (HH) and metabolic disorders remains to be fully elucidated. Several studies have documented that insulin resistance is the most important factor responsible for the association between low testosterone and T2DM ([15], for review), although conflicting results exist about the level—central and/or peripheral—at which the underlying pathogenic mechanisms may interfere with the HPG axis activity. By assessing insulin sensitivity with hyperinsulinemic euglycemic clamp, Pitteloud et al. [17] demonstrated that increased insulin resistance was associated with decreased Leydig cell testosterone secretion and not with LH pulses, thus indicating the implication of a peripheral impairment of the reproductive axis. A more recent study reported that patients with T2DM showed lower hypothalamic pulse frequency without changes in the pituitary response to GnRH nor testicular response to hCG [18]. Interestingly, in the same study it was reported that glucose levels were strongly correlated with the number of LH pulses, thus suggesting a specific negative effect of hyperglycemia in the hypothalamic secretion of GnRH [18]. In addition, hyperglycaemia has been identified as one of the major determinants for the association between metabolic syndrome (MetS) and hypogonadism [19]. A recent study in an animal model of high fat diet-induced MetS [20], aimed at investigating the contribution of the different metabolic derangements on the related HH condition, identified a strong association of reduced LH plasma levels with glucose intolerance severity, as well as with peculiar hypothalamic alterations, including increased expression of the glucose transporter GLUT4 [21]. These alterations occurred in the preoptic area of the hypothalamus, lining the third ventricle, where GnRH neurons reside and, accordingly, the same hypothalamic area was characterized by reduced immunopositivity for GnRH [20] and KISS1R [21]. Overall, these findings lead to further investigating the direct role specifically played by glucose in regulating GnRH neuron function.

To date, little is known about the direct effects of metabolic derangements on GnRH neurons in the human brain. In this study we took advantage of an *in vitro* model, the FNC-B4 cells, a long-term primary culture of human foetal GnRH-secreting neurons, obtained from a male fetus and previously characterized [22–24], which also express the kisspeptin/KiSS1R system [25, 26]. In order to investigate the direct effects of uncontrolled hyperglycemia on GnRH neurons, we exposed FNCB4 cells to elevated concentrations of glucose.

## 2. Materials and Methods

### 2.1. Cell Culture

The human GnRH-secreting FNC-B4 cells were established, cloned, and propagated *in vitro* from the olfactory system of a male fetus, cryogenically preserved, and previously characterized [22]. Cells were grown using Coon's modified F-12 medium (Irvine Scientific, Santa Ana, CA, USA) supplemented with 10% fetal bovine serum (Eurobio, Les Ulis, France) and antibiotic/antimycotic solution (penicillin, 100 IU/mL; streptomycin, 100 mg/mL). Before each experiment, cells at passages 5 to 10 were incubated with serum-free medium for 24 hours and then experiments were performed using glucose-free medium supplemented with either normal glucose (5 mM), high glucose (22 mM), very high glucose (40 mM), or mannitol (22 mM) for 24 hours. A subset of experiments was performed treating cells with 1 nM leptin for 24 hour in the presence of either 5 mM, 22 mM, or 40 mM glucose. Cells were washed in phosphate-buffered saline (PBS) and processed for quantitative mRNA expression or for immunocytochemistry procedures.

### 2.2. RNA Extraction and Quantitative RT-PCR

Isolation of RNA was performed using TRIZOL reagents according to manufacturer's instructions (Life Technologies Europe, Monza, Italy). cDNA synthesis was carried out using the iScriptTM cDNA Synthesis Kit purchased from Bio-Rad Laboratories (Hercules, CA). Quantitative RT-PCR (qRT-PCR) was performed with the fluorescent TaqMan methodology, as previously published [25], using specific primers and probe mixtures for GnRH1 (Hs00171272_m1), KISS1R (Hs00261399_m1), KISS1 (Hs00158486_m1), fibroblast growth factor receptor 1 (FGFR1; Hs00241111_m1), neuropilin 2 (NRP2; Hs00187290_m1), semaphorin 3A (SEMA3A; Hs00173810_m1), semaphorin 3F (SEMA3F; Hs00188273_m1), tachykinin 3 (TAC3; Hs00203109_m1), TAC3 receptor (TAC3R; Hs00357277_m1), androgen receptor (AR; Hs00171172_m1), estrogen receptor-*α* (ER*α*; Hs01046818_m1), ER*β* (Hs01100358_m1), G protein-coupled ER (GPER/GPR30; Hs00173506_m1), leptin receptor (LEPR; Hs00174497_m1), glucose transporter 1 (GLUT1; Hs00197884_m1), GLUT3 (Hs00359840_m1), and GLUT4 (Hs00168966_m1) mRNA, purchased from Life Technologies. The expression of the 18S ribosomal RNA subunit, chosen as the housekeeping gene, was quantified with a predeveloped assay (Hs99999901_s1; Life Technologies). Data analysis was based on the comparative threshold cycle (Ct) method, as previously described [27]. Amplification and detection were performed with the MyiQTM2 Two-Color Real-Time PCR Detection System (Bio-Rad Laboratories).

### 2.3. Immunocytochemistry

FNC-B4 cells were cultured on slides in the appropriate medium, then were fixed with 3.7% paraformaldehyde (pH 7.4) for 10 minutes and permeabilized with PBS containing 0.1% Triton X-100 (Sigma-Aldrich, St. Louis, MO) for 10 minutes. After rinsing in PBS, the slides were incubated with 1% bovine serum albumin for 15 minutes. Immunostaining was performed as previously described [25] using monoclonal anti-GnRH I antibody (1 : 200 dilution; Santa Cruz Biotechnology Inc.), rabbit polyclonal anti-kisspeptin (1 : 2000 dilution, Phoenix Pharmaceuticals, Inc., Belmont, CA), or rabbit polyclonal anti-KISS1R (1 : 100 dilution; Phoenix Pharmaceuticals, Inc.) followed by the conjugated antibodies: R6393 rhodamine red goat anti-mouse IgG (H + L) (1 : 200, Molecular Probes, Eugene, OR) for anti-GnRH I and A-11001 Alexa Fluor 488 goat anti-rabbit IgG (H + L) (1 : 200, Molecular Probes), for anti-kisspeptin or anti-KISS1R. The slides were evaluated and photographed using a Nikon Microphot-FXA microscope (Nikon). Immunopositivity quantification was performed using Photoshop 5.5 software (Adobe Systems Inc.).

### 2.4. Statistical Analysis

Data are expressed as the mean ± standard error of the mean (SEM) for n samples. Differences between more than two groups were assessed with one-way analysis of variance followed by Tukey-Kramer post hoc analysis. *P* < 0.05 was considered significant.

## 3. Results

**Figure 1:**
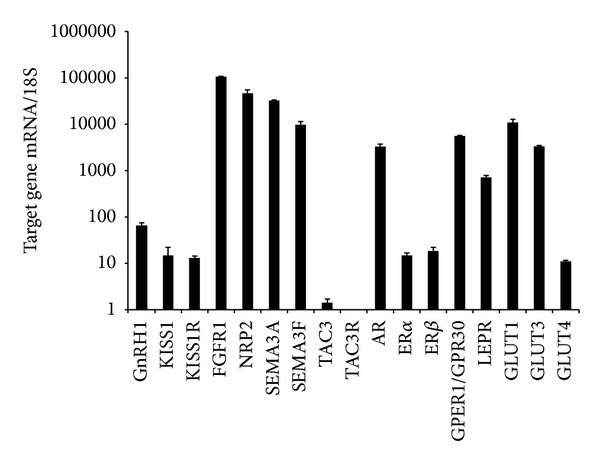
Gene expression profiling in FNC-B4 cells. Relative mRNA expression of genes relevant for GnRH neuron function was evaluated using quantitative RT-PCR. Data were calculated according to the comparative Ct method, using 18S rRNA subunit as the reference gene for normalization. Measurements were performed in four different cell preparations at different passages (from 5 to 10). No statistical differences were detected among passages, then results were pooled and reported as the mean ± SEM.

In order to provide a better characterization of the FNC-B4 cell phenotype, gene expression profiling was performed by quantitative RT-PCR in untreated cells. No differences were observed between the different passages (from 5 to 10), therefore, results were pooled and reported in Figure 1. FNC-B4 cells abundantly expressed GnRH and, at a lower extent, KISS1 and KISS1R mRNA. The most abundant expression was detected for genes known to be implicated in GnRH neuron migration, such as FGFR1, semaphorins (SEMA3A and SEMA3F), and their cognate receptor neuropilin 2 (NRP2). In contrast, TAC3 (otherwise known as neurokinin B) was almost undetectable and its cognate receptor (TAC3R) was not expressed by FNC-B4 cells. Interestingly, among the sex steroid receptors, AR and the G protein-coupled estrogen receptor GPER1/GPR30 were the most abundant, when compared to the classical ERs (ER*α* and ER*β*). Moreover, FNC-B4 cells expressed high levels of leptin receptor (LEPR), along with the glucose transporter isoforms GLUT1 and GLUT3. Although at a lower extent, FNC-B4 also expressed the insulin-dependent isoform GLUT4.

**Figure 2:**
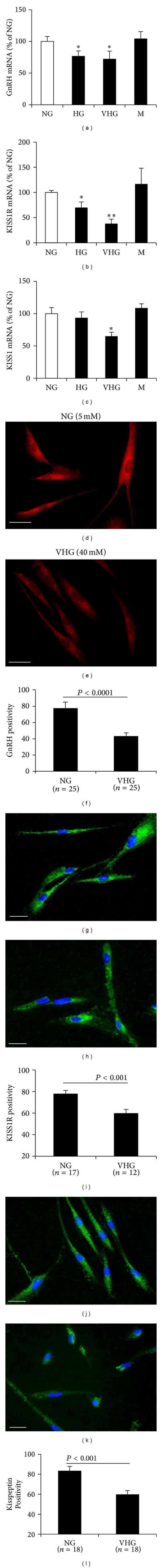
High glucose effects in FNCB4 cells. (a–c) Quantitative RT-PCR analysis of mRNA expression for GnRH (a), KISS1R (b) and KISS1, (c) genes in FNC-B4 cells exposed to normal (NG, 5 mM), high (HG, 22 mM), and very high (VHG, 40 mM) glucose concentration or mannitol (M, 22 mM) for 24 hours. Results were calculated according to the comparative Ct method, using 18S rRNA subunit as the reference gene for normalization and were obtained from three separate experiments, each performed in triplicate (*n* = 9). Data are reported as mean ± SEM and are expressed in percentage (%) of NG. *P < 0.05; **P < 0.01 versus NG. (d–l) Immunofluorescent localization of GnRH (d, and e), KISS1R (g and h), and kisspeptin (j and k) proteins in FNC-B4 cells exposed to NG (d, g, and j) or VHG (e, h, and k). Dual labeling with the nuclear staining DAPI (blue color) and anti-KISS1R (green color; g and h) or anti-kisspeptin (green color; j and k) antibodies is also shown. Original magnification ×20; scale bar = 50 *μ*m. Computer-assisted image analysis for quantification of GnRH, KISS1R, and kisspeptin immunopositivity is shown in panels (f), (i), and (l), respectively. *n* = number of analyzed cells.

To study whether the prolonged exposure to increasing glucose concentrations could interfere with the expression of genes related to GnRH neuron function, FNC-B4 cells were cultured for 24 hours in the presence of three different glucose concentrations (5 mM, 22 mM, and 40 mM). As shown in Figure 2, both high (22 mM) and very high (40 mM) glucose concentrations significantly reduced the mRNA expression of GnRH (Figure 2(a)) and KISS1R (Figure 2(b)), while KISS1 mRNA was inhibited only at the highest glucose concentration. Osmolarity-induced alterations were ruled out since no effects were observed by exposing cells to 22 mM mannitol (Figures 2(a)–2(c)). High glucose dependent down-regulation of GnRH, KISS1R, and KISS1 expression was also confirmed by immunocytochemistry analysis, as shown in Figure 2 by representative microphotographs (panels d-e, g-h, and j-k, resp.) and by the related computer-assisted quantification of immunopositivity intensity (panels f, i, and l, resp.).

**Figure 3:**
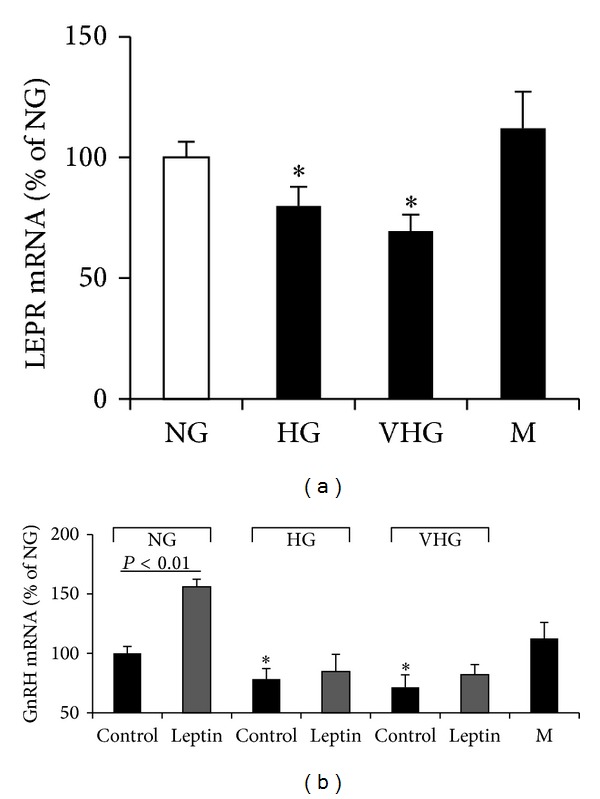
Effect of high glucose on leptin signaling in FNCB4 cells. (a) Quantitative RT-PCR analysis of LEPR mRNA expression in FNC-B4 cells exposed to normal (NG, 5 mM), high (HG, 22 mM), and very high (VHG, 40 mM) glucose concentration or mannitol (M, 22 mM) for 24 hours. (b) Effect of leptin (1 nM, 24 hours) on GnRH mRNA expression in FNC-B4 cells exposed to the different glucose concentrations (NG, HG, and VHG) or mannitol (M, 22 mM). Results were calculated according to the comparative Ct method, using 18S rRNA subunit as the reference gene for normalization and were obtained from at least three separate experiments, each performed in triplicate. Data are reported as mean ± SEM and are expressed in % of NG. *P < 0.05; **P < 0.01 versus NG.

The effects of high glucose exposure on GnRH expression were also studied in the presence of leptin, which is able to induce it in FNC-B4 cells, as previously demonstrated [26]. Accordingly, leptin treatment (1 nM, 24 hours) significantly increased GnRH mRNA expression in FNC-B4 cells at normal glucose (5 mM) (Figure 3(a)). However, this effect was lacking in the presence of high glucose concentrations (22 and 40 mM) (Figure 3(b)).

## 4. Discussion

Perturbation of glucose metabolism has been implicated as one of the pathogenic factors responsible for the association between HH and metabolic disorders [19]. In experimental MetS, hyperglycemia and related hypothalamic inflammatory processes have been associated with the impairment of GnRH/gonadotropin release [20]. Using a well-characterized cellular model, we here demonstrate a direct inhibitory action of increasing glucose concentrations on human foetal GnRH-secreting neurons, the FNC-B4 cells, thus unraveling that under pathological conditions, high levels of glucose may directly inhibit the expression of genes relevant for GnRH neuron function.

It is well known that glucose is a key metabolic regulator of the reproductive axis, able to fine-tune pulsatile GnRH release ([28], for review). The study by Herde et al. [4], showing that subpopulations of GnRH neurons may direct sense from the periphery, greatly improved our understanding of how abrupt changes in the plasma level of molecules can modulate pulsatile GnRH/LH secretion. Recent evidences in the mouse GT1-7 clonal GnRH cell line have implicated this GnRH neuronal cell line as direct sensor of glucose, further suggesting that GnRH neurons may sense changes in extracellular glucose directly [29, 30] and that this glucosensing is modulated by gonadal steroids [29, 31]. However, the majority of existing evidence for glucose regulation of GnRH neuron activity is derived from studies of experimental glucoprivation, showing that GnRH neurons are sensitive to changes in glucose concentrations within the physiological range (up to 5 mM), with low doses (<0.5 mM) being able to downregulate GnRH release [29, 31, 32]. In this study, we have tested the effects of exposing FNC-B4 cells to extremely high glucose concentrations (22 and 40 mM), in order to mimic pathological conditions, such as uncontrolled diabetic hyperglycemia, when brain glucose levels more likely exceed 5 mM.

The investigation of GnRH neuron biology is strongly hampered by the peculiar anatomical distribution of these few hundreds of cells scattered within the hypothalamic POA. The availability of FNC-B4 cells have facilitated the study of mechanisms regulating GnRH neurons of human origin [22–26, 33–36]. These cells express and release GnRH in response to different stimuli, including sex steroids [23], kisspeptin [25], and leptin [26]. Moreover, previous studies have identified that FNC-B4 cells express the insulin growth factor (IGF) system which was downregulated by high glucose (20 mM) exposure [37]. We here show that FNC-B4 cells express glucose transporters (GLUT1, GLUT3, and GLUT4) and further demonstrate that these cells may respond to changes in glucose concentrations, thus opening new mechanistic insights into the direct metabolic control of GnRH release. Exposing FNC-B4 cells to high glucose significantly reduced gene and protein expression not only of GnRH but also KISS1R, which, upon activation by kisspeptin, is regarded as the master regulator of GnRH production. As previously demonstrated [25], we here confirm that FNC-B4 cells express KISS1, whose expression is also impaired by high glucose exposure. This finding is apparently in contrast with the current understanding of KISS1/KISS1R system in the forebrain of the mouse and rat, which indicates that kisspeptin-secreting neurons are a hypothalamic neuronal subpopulation distinct from that of GnRH-secreting neurons ([8], for review). However, in good agreement with our results, the mouse GT1-7 cell line, which are immortalized hypothalamic GnRH neurons [38], express KISS1 [39]. Gene expression profiling better clarified the FNC-B4 phenotype, which abundantly express genes, such as FGFR1, NRP2, SEMA3A, and SEMA3F, known to be involved in the normal migratory processes, through which GnRH neurons reach the final hypothalamic destination during embryogenesis. It is well known that FGFR1 mutations cause the Kallmann syndrome, a heterogeneous genetic disorder that associates HH due to GnRH deficiency with anosmia [40]. Similarly, neuropilins (NRP1 and NRP2) and their ligands semaphorins (SEMA3A and SEMA3F) have been implicated in the development of the GnRH system [41]. Interestingly, FNC-B4 cells not only express the classical androgen and estrogen receptors (AR, ER*α*, and ER*β*), as previously reported [23, 26], but also the membrane estrogen receptor GPER1/GPR30, which has been recently implicated in rapid action of estrogen both in primates [42] and mice [43] GnRH neurons. Moreover, the identity of FNC-B4 cells as GnRH neurons is corroborated by the observation that these cells do not express TAC3, the gene encoding the neurokinin B (NKB), nor do they express TAC3R, which encodes for NKB receptor. This finding is in agreement with studies reporting the lack of TAC3R in GnRH neurons of sheep [44, 45] and mice [46]. Indeed, it has been assumed that that NKB is released by KISS1 neurons and not by GnRH neurons and acts to enhance kisspeptin secretion by an autocrine or paracrine mechanism through TAC3R [47]. Accordingly, kisspeptin infusion restores gonadotropin pulsatility in patients with TAC3 or TAC3R loss-of-function mutations [47].

In this study, we originally demonstrated the regulation of leptin signalling in FNC-B4 cells by glucose exposure. The role of leptin as permissive metabolic signal for reproduction, acting through a stimulatory effect of the hormone on GnRH secretion is well known [48]. As previously demonstrated [26], we here confirm that at 5 mM glucose concentration leptin retained the ability of inducing GnRH expression in FNC-B4 cells. In contrast, leptin failed to stimulate GnRH expression in the presence of high glucose doses, thus suggesting an impaired leptin signalling, most likely due to the inhibitory effect of both 22 and 40 mM glucose concentrations on LEPR expression. Although it is generally accepted that GnRH neurons do not physiologically express LEPR, suggesting the involvement of intermediate neuronal circuits and signals [49], our results are in agreement with previous reports showing that the stimulatory effect of leptin on GnRH secretion may be direct on cells, which, similarly to FNC-B4, express LEPR [50].

Since FNC-B4 cells have a male karyotype, any extrapolation of our results to explain the control of GnRH neurons by glucose is limited to male patients. Indeed, the effects of MetS on the reproductive female system (increased secretion of LH, hyperandrogenism) are quite different from those occurring in males. Sexual dimorphism of hypothalamic nuclei, which in females are involved in mediating the positive feedback of ovarian steroids, essential for the preovulatory luteinizing hormone surge, could be implicated in the different response of the female reproductive system to metabolic disorders. Further studies could clarify whether neurons, similar to FNC-B4, with a female karyotype would have a different control of the same genes and proteins.

In conclusion, even if obtained *in vitro*, our findings support the idea of a deleterious direct contribution of hyperglycemia on human GnRH neurons, thus providing new insights into the pathogenic mechanisms linking HH to metabolic disorders.
